# Knowledge, Attitudes, and Practices toward Antimicrobial Resistance among Young Italian Nurses and Students: A Multicenter, Cross-Sectional Study

**DOI:** 10.5334/aogh.4488

**Published:** 2024-07-22

**Authors:** Elda De Vita, Francesco Vladimiro Segala, Luisa Frallonardo, Giovanni Civile, Denise De Scisciolo, Roberta Novara, Andrea De Vito, Maria Giacobba De Girolamo, Angela Amendolara, Luigi Piccolomo, Giordano Madeddu, Antonio Terranova, Davide Mariani, Salvatore Altavilla, Nicola Veronese, Mario Barbagallo, Giancarlo Cicolini, Francesco Di Gennaro, Annalisa Saracino

**Affiliations:** 1 Clinic of Infectious Diseases, Department of Precision and Regenerative Medicine and Ionian Area (DiMePRe-J), University of Bari “Aldo Moro,” 70124 Bari, Italy; 2 Department of Biomedicine and Prevention, Tor Vergata University, Rome, Italy; 3 Department of Precision and Regenerative Medicine and Ionian Area-(DiMePRe-J), University of Bari “Aldo Moro,” 70124 Bari, Italy; 4 Geriatric Unit, Department of Medicine, University of Palermo, 90100 Palermo, Italy; 5 Unit of Infectious Diseases, Department of Medicine, Surgery, and Pharmacy, University of Sassari, Sassari 07100, Italy

**Keywords:** antimicrobial resistance (AMR), infection control, health professional, antimicrobial stewardship (AMS), survey

## Abstract

*Background:* Nurses play a pivotal role in combating antimicrobial resistance (AMR). However, the success of local and national AMR containment efforts hinges on the knowledge, attitude, and practice (KAP) of nursing staff and undergraduate students.

*Objectives:* This study aims to explore the determinants of nurses’ KAP regarding AMR, offering insights to control the emergence and spread of drug-resistant pathogens.

*Methods:* This cross-sectional, multicenter survey involving Italian nurses, nursing students, and healthcare professionals was conducted administering an anonymous online questionnaire focusing on AMR. The median score of 12 was taken as the cutoff for “good KAP.” The association between study variables and good KAP was assessed using chi-square or t-tests, followed by multivariable logistic regression analysis for statistically significant (*p* < 0.05) variables.

*Findings:* Among 848 participants, 61.9% (*n* = 525) were students, and 39.6% (*n* = 336) scored as having “low KAP.” High KAP was associated with being female and studying AMR independently. Conversely, living in southern Italy and receiving AMR training from pharmaceutical companies were associated with low KAP.

*Conclusions:* Among Italian nurses, AMR awareness relies on those who have studied AMR as self-taught and is affected by gender and region. Italian universities lack in lectures on AMR management, and much needs to be done to improve awareness of antimicrobial stewardship among nonmedical health workers.

## 1. Introduction

Antimicrobial stewardship (AMS) involves systematic actions aimed at improving patient outcomes, limiting adverse events, and curbing the emergence and spread of antimicrobial resistance (AMR) [1]. In the Council Recommendation of the European Centre for Disease Prevention and Control (ECDC) to face antimicrobial resistance, an EU target on total antibiotic consumption to be reached by 2030 is set with a One Health approach. However, antimicrobial consumption (AMC) data of 2022 were worrying, with half of the ECDC member states, including Italy, having AMC values above the EU mean consumption rate in 2019 and even higher AMC rates in 2022—unlike from Finland, the Netherlands, and Sweden, which had already reached their recommended target AMC level for 2030 in 2022 [2]. Since 2015, when the 68th World Health Assembly (WHA) approved the Global Action Plan (GAP) on Antimicrobial Resistance (AMR), the World Health Organization (WHO) invited countries to formally commit themselves to addressing AMR [3]. To face this challenge a multifaceted strategy is needed, with a diversified approach including improvements in clinical practice, a proper use of antimicrobial agents, and strategies to increase the knowledge of AMR [4].

As largely demonstrated in the literature, implementing national strategies to limit AMR can be challenging and time-consuming without a multidisciplinary approach. However, AMS predominantly remains a domain of doctors and pharmacists [5]. In this regard, nurses, who play a pivotal role in patient management, have been shown to impact clinical outcomes significantly [6]. Being involved in every phase of patient care, from admission to discharge, together with doctors and other healthcare professionals, nurses are often the first line of defense [7], with a potential key role in reducing the spread of colonization and infections by managing an appropriate antimicrobial strategy [8].

Furthermore, as self-medication and patient expectations from medicaments are some of the leading causes of AMR [9], nurses would play a crucial role in patient education [6], guiding them toward a more conscious use of antimicrobial agents [10].

In this regard, as stated by Hamdy et al. in 2019 [11], nurses have the possibility to identify and bridge communication gaps between doctors and patients or their families, explaining the use and risks of antimicrobials. Additionally, nurses feel often capable of acquiring knowledge about antimicrobial use, becoming passionate and proactive about antimicrobial use, expressing a strong desire to enhance their knowledge, and being motivated to have a role in AMS, although perceiving a lack of educational focus on antimicrobials within nursing practice [12]. In this regard, it has been found globally that nurses feel inadequately prepared to participate in antimicrobial stewardship due to a lack of education and supportive hospital culture [13]. In this aspect, the AMR EDUCare project highlights a lack of implementation of the educational aspects of national action plans on AMR with a disparity in the availability of AMR education among healthcare workers, including nurses, in many European countries, including Italy [14]. This has lead to a lack of recognition of their position in the management of AMR [15], which tends to focus on physician- and pharmacist-led stewardship interventions. Additionally, AMR educational programs rarely target nurses, and this oversight is relevant, as a lack of understanding of AMS principles and a lack of knowledge of proper antimicrobial use may affect prevention and control strategies. Recent studies have highlighted that the spread of antibiotic resistance was higher in settings where there was a limited awareness and knowledge regarding the use of antibiotics [16]. By adhering to educational strategies, guidelines, and protocols, healthcare professionals can effectively reduce the risk of AMR and improve patient outcomes through better disinfection and antimicrobial stewardship practices. Educational programs on AMR for healthcare professionals can be delivered through training programs, workshops and seminars with interactive sessions (including case studies, role-playing, and group discussions [17]), as well as through refresher courses following IDSA (Infectious Diseases Society of America) and WHO guidelines and good clinical practice protocols for disinfection and AMS, focusing on practical aspects such as practice protocols for disinfection and the five moments of hand hygiene [18].

These findings underscore the distance yet to be covered to enhance local and national AMR containment efforts by improving the KAP of nursing staff.

The present study aims to explore the determinants of nurses’ KAP regarding AMR in an Italian multicenter study, offering insights for tailored interventions to prevent the emergence and spread of drug-resistant pathogens.

## 2. Materials and Methods

### 2.1 Study design, setting, and population

This is a cross-sectional, multicenter survey conducted from 21 October 2022 to 12 June 2023. An anonymous online questionnaire was administered to Italian nurses, nursing students, and other healthcare professionals, regardless of the setting or region in which they were working. For recruitment, we employed convenience sampling, and no exclusion criteria were applied in this study. The study followed STROBE (Strengthening the Reporting of Observational Studies in Epidemiology) guidelines for observational studies [19]. Data were collected and managed using the REDCap mobile app electronic data capture tool hosted at Catholic University of the Sacred Heart, Rome.

### 2.2 Questionnaire structure

Data were collected by administering a structured questionnaire consisting of 58 close-ended questions divided into 3 parts. The first section gathered personal information (e.g., age, sex, education), the second part evaluated AMR awareness, and the third part focused on attitudes and practices.

### 2.3 Study endpoints

The primary endpoint of this study was to assess the KAP scores related to AMR among nursing students, nurses, and other healthcare professionals. Secondary endpoints included evaluating the association between demographic and professional characteristics (e.g., gender, geographical location, and source of AMR training) and the KAP scores, as well as identifying variables that significantly influence the KAP scores.

### 2.4 Statistical analysis

A descriptive analysis was performed to define the distribution of baseline variables and characteristics of the sample. The dependent variable was a good AMR KAP, and a score higher than the median result (12 points) was considered “good knowledge” of AMR. Continuous variables were compared between groups using the Mann-Whitney U test, while categorical variables were analyzed using a chi-squared test. A logistic regression model was used, with AMR good knowledge as the dependent variable and the available factors at the baseline evaluation as independent variables in the univariate analysis. Variables found to be significant (*p*-value < 0.05) in the univariate analysis were included in the multivariate analysis. The strength of the association between baseline factors and good AMR CAP (outcome) was measured using adjusted odds ratios (aORs) with 95% confidence intervals (CIs). All statistical tests were two-tailed, and a *p*-value < 0.05 was considered statistically significant. Statistical analyses were performed using R Statistical Software (v4.1.3; R Core Team 2021) in R Studio Version.

### 2.5 Ethical approval

Ethical approval was not required; participation was voluntary, anonymous, without compensation, and did not involve the collection of clinical data. Informed consent for completion of the questionnaire was declared on the first page. However, the study was conducted in accordance with the Declaration of Helsinki and national and institutional standards.

## 3. Results

**Figure 1:**
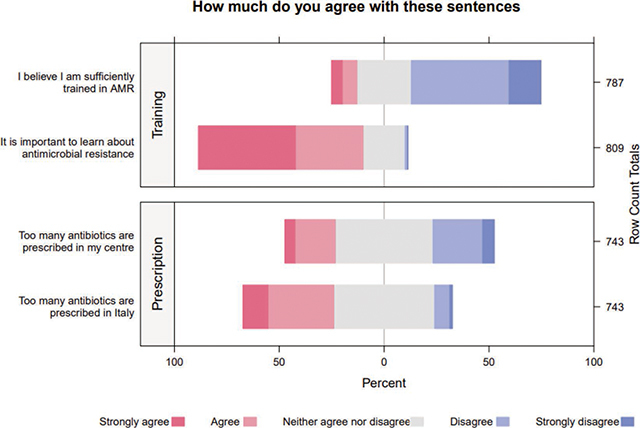
Likert Scale of Training on AMR and Personal Beliefs about the Prescription of Antibiotics.

As shown in Table 1, a total of 848 nursing students, nurses, and other healthcare professionals participated in the survey. Among them 61.9% (*n* = 525) were students, while 37.3% (*n* = 316) were registered nurses. The majority of the population (71.58%, *n* = 607) identified as female, and the median age was 22 (IQR 21–25), with 82.3% (*n* = 698) residing in southern Italy. Most of the interviewed were students (61.9%, = 525), with 55.9% of them attending second or third year of university, and 39.6% (*n* = 336) scored as having “low KAP,” with a median of the years of service of 6.50 (IQR 3–11.5). Figure 1 show the response to selected questions.

**Table 1:** Descriptive characteristics of 848 participants stratifying for high or low knowledge of AMR.

	OVERALL (*N* = 848)	HIGH KAP^2^ (*N* = 512)	LOW KAP^3^ (*N* = 336)	*P*-VALUE
**Which gender do you identify with?**				
Female	607 (71.6%)	393 (76.8%)	214 (63.7%)	**<0.001**
Male	229 (27.0%)	114 (22.3%)	115 (34.2%)	
Nonbinary	4 (0.5%)	3 (0.6%)	1 (0.3%)	
Other	2 (0.2%)	0 (0%)	2 (0.6%)	
Missing	6 (0.7%)	2 (0.4%)	4 (1.2%)	
**Age**				
Median [Q1, Q3]	22.0 [21.0, 25.0]	22.0 [21.0, 26.0]	22.0 [21.0, 24.0]	**0.0014**
Missing	14 (1.7%)	6 (1.2%)	8 (2.4%)	
**Current position**				
Working	316 (37.3%)	217 (42.4%)	99 (29.5%)	**<0.001**
Studying	525 (61.9%)	294 (57.4%)	231 (68.8%)	
Missing	7 (0.8%)	1 (0.2%)	6 (1.8%)	
**Area**				
Central Italy	58 (6.8%)	47 (9.2%)	11 (3.3%)	**0.00424**
Northern Italy	71 (8.4%)	44 (8.6%)	27 (8.0%)	
Southern Italy	698 (82.3%)	412 (80.5%)	286 (85.1%)	
Missing	21 (2.5%)	9 (1.8%)	12 (3.6%)	
**Profession**				
Nurse	307/316 (97.2%)	213/316 (67.4%)	94/316 (29.7%)	-
Healthcare profession	8 (0.9%)	3 (0.6%)	5 (1.5%)	
**Bachelor’s degree**				
Other	2 (0.2%)	1 (0.2%)	1 (0.3%)	0.984
Nursing	516 (60.8%)	289 (56.4%)	227 (67.6%)	
Obstetrics (midwife)	7 (0.8%)	4 (0.8%)	3 (0.9%)	
Missing	323 (38.1%)	218 (42.6%)	105 (31.3%)	
**Setting**				
Other services	209 (24.6%)	125 (24.4%)	84 (25.0%)	0.808
Ambulatory/DH	6 (0.7%)	5 (1.0%)	1 (0.3%)	
Intensive care	35 (4.1%)	24 (4.7%)	11 (3.3%)	
Obstetrics	23 (2.7%)	16 (3.1%)	7 (2.1%)	
Emergency room	54 (6.4%)	31 (6.1%)	23 (6.8%)	
Rehabilitation	12 (1.4%)	7 (1.4%)	5 (1.5%)	
Surgical setting	171 (20.2%)	101 (19.7%)	70 (20.8%)	
Clinical setting	240 (28.3%)	150 (29.3%)	90 (26.8%)	
Missing	98 (11.6%)	53 (10.4%)	45 (13.4%)	
**Academic year**				
First year	45 (5.3%)	9 (1.8%)	36 (10.7%)	**<0.001**
Second year	282 (33.3%)	180 (35.2%)	102 (30.4%)	
Third year	192 (22.6%)	101 (19.7%)	91 (27.1%)	
Missing	329 (38.8%)	222 (43.4%)	107 (31.8%)	
**Years of service**				
Median [Q1, Q3]	5.00 [2.50, 12.0]	5.00 [2.00, 12.0]	6.50 [3.00, 11.5]	0.27
Missing	533 (62.9%)	296 (57.8%)	237 (70.5%)	
**Did you receive any education on AMR from your hospital/university? Answer: “Yes”**	156 (18.4%)	101 (19.7%)	55 (16.4%)	0.253
**How were you trained on the subject of AMR?**				
Self taught	315 (37.1%)	216 (42.2%)	99 (29.5%)	**<0.001**
Master courses	24 (2.8%)	20 (3.9%)	4 (1.2%)	**0.0339**
University	519 (61.2%)	319 (62.3%)	200 (59.5%)	0.459
Events funded by pharmaceutical companies?	8 (0.9%)	1 (0.2%)	7 (2.1%)	**0.0156**
Hospital meetings	320 (37.7%)	195 (38.1%)	125 (37.2%)	0.852
Hospital/University meetings with external lecturers	36 (4.2%)	25 (4.9%)	11 (3.3%)	0.336

^1^ Bold The *p*-value represents a statistically significant variable.

^2^ High knowledge, attitude, and practice (KAP) scores related to antimicrobial resistance (AMR).

^3^ Low knowledge, attitude, and practice (KAP) scores related to antimicrobial resistance (AMR).

In the multivariate analysis (Table 2), being female (aOR 1.69, 95% CI 1.23–2.34; *p* = 0.001) and independently studying AMR (aOR 1.53, 95% CI 1.12–2.11; *p* = 0.007) were variables associated with a high KAP score. In contrast, residing in southern Italy (aOR 0.47, 95% CI 0.22–0.96; *p* = 0.04) and receiving AMR training from pharmaceutical companies (aOR 0.08, 95% CI 0.004–0.531; *p* = 0.02) were associated with a low KAP score.

**Table 2:** Crude multiple logistic regression for factors associated with high KAP.

FACTOR	AOR	LOW: 95%CI	HIGH: 95%CI	*P*-VALUE
(reference: Males)	0.906	0.507	1.636	0.740
Females	1.699	1.232	2.344	**0.001**
Being a student	0.717	0.500	1.022	0.068
Living in Central Italy	2.507	1.098	6.029	**0.033**
Living in Southern Italy	0.479	0.220	0.962	**0.048**
Being self-taught	1.538	1.126	2.110	**0.007**
Having attained a Master class on AMR	2.954	1.020	11.169	0.068
Working or studying in a setting where AMR training is provided by pharmaceutical companies.	0.082	0.004	0.531	**0.027**

aOR: adjusted odds ratio; CI: confidence interval; bold *p*-value represents a statistical significative variable.

## 4. Discussion

In recent years, nurses are increasingly recognized as key actors in antimicrobial stewardship (AMS) programs due to their unique position within healthcare teams and their direct interactions with patients. Their involvement is crucial in ensuring the appropriate use of antibiotics, which helps in reducing antibiotic resistance, improving patient outcomes, and optimizing healthcare resources [20, 21], with increasing efforts being made to broaden the knowledge in this area [22]. In our study, being female and having studied AMR as an autodidact were associated with high KAP score, while being trained during events funded by pharmaceutical companies and living in southern Italy were linked to low KAP score; however, the latter should be considered as a bias as 85.3 % of enrolled participants worked or studied in southern Italy, with Apulia as the most represented region (53% of the interviewed) (Table 1).

Nurses often exhibit a higher level of awareness about the national action plan on antibiotic resistance, proving to be more capable of adhering to WHO’s five moments of hand hygiene [18] while having the lowest knowledge about antimicrobic use and resistance [23]. A study conducted in Italy in early 2020 demonstrated that also among young doctors, the percentage of correct answers on multidrug-resistant germs was very low, despite most claiming to have a good knowledge on AMR [24]; the same results have been reached by many other studies conducted among under- and post-graduate medical doctors [25, 26]. By contrast, in the present study, most of the participants felt they lacked sufficient AMR training (Figure 1); nevertheless, the highest percentage of correct answers was given in the Knowledge section, while the lowest score was obtained in the Practices section (mean score 6.7 (± 1.4) vs. 4.1 (± 2.3)).

In the Attitude section, it is worth stressing that only 15.7% of the participants agreed with the statement “I dedicate time to educate patients, caregivers, colleagues, support staff about infection risks, mitigation, and AMR management” (Table 3). The relatively high scores in terms of knowledge among all participants can likely be attributed to their young age and recent completion of university studies. However, the lack of experience in clinical practice may have hindered certain skills, such as ensuring that urine bags are not lifted off the ground (Table 3). As expected, the worker group exhibited a higher practice score than the students (4.3 ± 2.1 vs. 4.0 ± 2.4).

**Table 3:** Composition of the outcome score.

ITEM	WORKERS (*N* = 316)	STUDENTS (*N* = 525)	OVERALL (*N* = 841)	*P*-VALUE
**Knowledge**				
The optimal intravenous infusion time for Piperacillin/Tazobactam is 3 hours/continuous infusion	152 (48.1%)	212 (40.4%)	364 (43.3%)	0.034
Wearing gloves replaces handwashing	302 (95.6%)	510 (97.1%)	812 (96.6%)	0.31
The use of hand sanitizer is equivalent to washing hands with soap and water	204 (64.6%)	369 (70.3%)	573 (68.1%)	0.098
The COVID-19 pandemic has reduced the spread of AMR in our country	248 (78.5%)	369 (70.3%)	617 (73.4%)	0.011
It is good practice for all hospitalized patients to be under antibiotic coverage	296 (93.7%)	419 (79.8%)	715 (85.0%)	<0.001
In a patient in septic shock, antibiotic administration is one of the actions to be taken during the ‘golden hour’	235 (74.4%)	345 (65.7%)	580 (69.0%)	0.010
Which of these procedures is NOT part of the 5 fundamental moments of Hand Hygiene: wash your own hands:	208 (65.8%)	279 (53.1%)	487 (57.9%)	<0.001
Antibiotic resistance is an expressed property	223 (70.6%)	385 (73.3%)	608 (72.3%)	0.431
KNOWLEDGE SCORE - Mean (SD)	6.9 (± 1.5)	6.5 (± 1.4)	6.7 (± 1.4)	<0.001
**Attitudes**				
How willing are you to support a specific Antibiotic Resistance exam during your academic path?	268 (84.8%)	384 (73.1%)	652 (77.5%)	<0.001
How willing are you to attend AMR prevention courses?	292 (92.4%)	451 (85.9%)	743 (88.3%)	0.006
How willing are you to create a monitoring network for correct antibiotic administration and report any resistance cases?	291 (92.1%)	437 (83.2%)	728 (86.6%)	<0.001
How willing are you to follow indications and procedures that reduce antibiotic resistance?	300 (94.9%)	474 (90.3%)	774 (92.0%)	0.022
ATTITUDE SCORE - Mean (SD)	4.6 (± 0.79)	4.3 (± 1.1)	4.4 (± 1.0)	<0.001
**Practices**				
I wash my hands before putting on gloves	183 (57.9%)	276 (52.6%)	459 (54.6%)	0.151
I wash my hands after removing gloves	221 (69.9%)	326 (62.1%)	547 (65.0%)	0.025
I adhere to the antibiotic administration timeframes	133 (42.1%)	254 (48.4%)	387 (46.0%)	0.088
I use disposable gowns in contact isolations	175 (55.4%)	243 (46.3%)	418 (49.7%)	0.013
I notice that contact isolations are not adequately indicated	55 (17.4%)	112 (21.3%)	167 (19.9%)	0.196
I agree that the provided materials (gloves, hand sanitizer, disposable gowns, ROT, etc.) are not readily available	46 (14.6%)	95 (18.1%)	141 (16.8%)	0.217
I ensure that the urine bag in patients with urinary catheters is lifted off the ground	174 (55.1%)	191 (36.4%)	365 (43.4%)	<0.001
I dedicate time to educate patients, caregivers, colleagues, support staff about infection risks, mitigation, and AMR management	46 (14.6%)	86 (16.4%)	132 (15.7%)	0.544
**Practices score** - Mean (SD)	4.3 (± 2.1)	4.0 (± 2.4)	4.1 (± 2.3)	0.0591
**KAP score** Mean (SD)	14 (± 3.0)	13 (± 3.2)	13 (± 3.2)	<0.001

The table shows the total number and percentage of correct answers. Bold p value represents a statistical significative variable.

Furthermore, in the knowledge section, almost 74% of the respondents agreed with the sentence “The COVID-19 pandemic has reduced the spread of AMR in our country.” Globally, the COVID-19 pandemic led to increased antimicrobial use [27] due to the concern of bacterial co- and superinfections [28], contributing to the spread of multidrug-resistant strains [29]. In particular, during pandemic peaks, there are concerns that the spread of biofilm-producing pathogens such as *A. baumannii* and *Staphylococcus aureus* [30] may have been enhanced by the loss of key infection control practices, such as changing or disinfecting gloves when passing from one patient to another. The COVID-19 pandemic has significantly impacted perceptions of AMR. The increased use of antibiotics to treat COVID-19 patients, especially early in the pandemic when the clinical understanding of the virus was still developing, has fueled concerns about AMR. Many COVID-19 patients received antibiotics to treat secondary bacterial infections or out of precaution; this overuse, with the potential to accelerate the development of resistant bacteria​, has collectively heightened the urgency to address AMR through improved detection, characterization, and response strategies [31].

In this regard, despite having shown a high capacity for adaptation and a high level of competence in the pandemic [32], during which the role of nurses has been largely glorified by mass media [7], specific AMS interventions targeting or involving nurses were lacking.

Another peculiar finding is that receiving AMR training from pharmaceutical companies correlated with low KAP scores. However, it is crucial to note that only the 1.9 % (*N* = 16) of the interviewed declared such things, while the majority were trained at university (61.20% [*N* = 519]) or self-taught (37.14% [*N* = 315]) (Table 3); this last variable was also associated with a high KAP score (aOR 1.53, 95% CI 1.12–2.11; *p* = 0.007).

Effective AMR management requires contextualized programs. To enhance nurses’ and other health professionals’ performance, educational strategies aiming to improve self-awareness should be evaluated. In a good AMR monitoring and management program, contextualization is the keystone for a good outcome. It should be noted that in our study while many health professionals were aware of inappropriate antimicrobial prescriptions in Italy, they did not recognize such practices within their own centers. In addition, 86.7% (*N* = 735) declared not to know a colleague with a specific training on AMR or infection control, and they disagreed that they were sufficiently trained in AMR (Figure 1). Furthermore, only 46% declared to adhere to the antibiotic administration timeframes, and only half of the interviewed (49.7%) declared to use disposable gowns in contact isolations, despite almost all of them declaring that provided materials were available (Table 3). These data are in line with other studies in the literature [33], suggesting that despite personal equipment being present, there is a poor use of devices by the nursing staff. Therefore, to boost the performance of nurses and health professionals, educational strategies in enhancing self-awareness must be evaluated [34].

One of the most important determinants of the emergence of antibiotic resistance is poor training for healthcare professionals [35, 36]. A multicounty study conducted in 2022 clearly demonstrated that nurses with AMS training exhibited higher AMS-related skills, in terms of capability and motivation to perform AMS, emphasizing the impact of quality training on AMS behaviors [37]. Much needs to be done in reducing AMR by improving hospital staff performance and reducing the theory–practice gap [38]. Strategies should focus on continuous education on AMR and AMS, with continuous training and periodical evaluations on knowledge and skills, possibly integrating them into academic curricula and/or specialized degree programs.

Limitations of this study were several: first, the young age of the participants does not give the right representation of the population, considering that the average age of nurses in Italy is 52.2 years [39]. Furthermore, the study recruitment was conducted starting from the region Apulia, and the sample is not representative of the entire country; since, as previously stressed, 53% of the interviewed were from this region, the lack of randomization during the recruitment does not give a faithful view of other contexts. Similarly to other cross-sectional surveys [40], our study represents a single snapshot in a multifaceted panorama, in which evidence and perceptions may vary. Finally, the study relied on self-reported data, which may be subject to social desirability bias.

On the other hand, strengths of the study included the multicenter approach, the relatively high numerosity, the use of a robust data collection tool (REDCap), and the use of a structured questionnaire following standardized guidelines.

## 5. Conclusions

Good knowledge, attitudes, and practices related to AMR among nurses were associated with being female, working in Central Italy, and relying on self-teaching. Participants demonstrated a positive attitude toward adherence to guidelines and a willingness to engage in further education focused on AMR prevention, indicating a readiness to enhance their professional competencies to combat AMR effectively. However, the study also unveiled concerning practices, such as inconsistencies in the use of disposable gowns in contact isolations and adherence to antibiotic administration timeframes, highlighting areas that necessitate immediate improvement.

Our study offers insights for tailored interventions to prevent the emergence and spread of drug-resistant pathogens, such as an effective training during university years and continuous refresher courses for nurses and hospital staff. The emphasis should be on a multifaceted educational approach that includes active involvement in decision-making processes, patient education, and adherence to best practices in infection control and antibiotic administration. Participants in the study demonstrated a positive attitude toward adherence to guidelines and a willingness to engage in further education focused on AMR prevention. This indicates a readiness to enhance their professional competencies to combat AMR effectively. The evolving landscape of healthcare in 2024 necessitates that healthcare professionals possess a skill set far more diverse and specialized than that of their predecessors from 20–30 years ago. Modern medical and nurse training must now emphasize infection control, climate change, and AMR as essential components. Medical and nurse faculties need to adapt their curricula to provide contemporary and comprehensive training in these areas. By doing so, they will ensure that future healthcare professionals are equipped to handle the complex health challenges of our time [41].

## Data Availability

All data generated or analyzed during this study are included in this published article [and its supplementary information files].

## Public Involvement Statement

No public involvement in any aspect of this research.

## Guidelines and Standards Statement

The manuscript followed STROBE guidelines for observational studies.

## Additional File

The additional file for this article can be found as follows:

Supplementary Table. Distribution of KAP correct answers stratified by job position.

## References

[r1] Majumder MAA, Rahman S, Cohall D, et al. Antimicrobial stewardship: Fantimicrobial resistance and protecting global public health. Infect Drug Resist. 2020;13:4713–4738. doi:10.2147/IDR.S290835. 33402841 PMC7778387

[r2] European Centre for Disease Prevention and Control. Antimicrobial consumption in the EU/EEA (ESAC-Net) - Annual Epidemiological Report for 2022. Retrieved from https://www.ecdc.europa.eu/en/publications-data/antimicrobial-consumption-eueea-annual-epidemiological-report-2022.

[r3] WHO. Comprehensive Review of the WHO Global Action Plan on Antimicrobial Resistance-Volume 1: Report. https://www.who.int/publications/m/item/comprehensive-review-of-the-who-global-action-plan-on-antimicrobial-resistance. Accessed March 11, 2024.

[r4] Castro-Sánchez E, Gilchrist M, Ahmad R, Courtenay M, Bosanquet J, Holmes AH. Nurse roles in antimicrobial stewardship: Lessons from public sectors models of acute care service delivery in the United Kingdom. Antimicrob Resist Infect Control. 2019;8:162. doi:10.1186/s13756-019-0621-4. 31649819 PMC6805549

[r5] Sutthiruk N, Considine J, Hutchinson A, Driscoll A, Malathum K, Botti M. Thai clinicians’ attitudes toward antimicrobial stewardship programs. Am J Infect Control. 2018;46:425–430. doi:10.1016/j.ajic.2017.09.022. 29132695

[r6] Wells-Federman C, Arnstein P, Caudill M. Nurse-led pain management program: Effect on self-efficacy, pain intensity, pain-related disability, and depressive symptoms in chronic pain patients. Pain Manag Nurs. 2002;3:131–140. doi:10.1053/jpmn.2002.127178. 12454805

[r7] Rossi S, Cosentino C, Bettinaglio GC, et al. Perception of nurses’ professional identity during the first wave of COVID-19 pandemic infections. Acta Biomed. 2021;92:e2021036. doi:10.23750/abm.v92iS2.11959. PMC8383219 34328129

[r8] Davey K, Aveyard H. Nurses’ perceptions of their role in antimicrobial stewardship within the hospital environment: An integrative literature review. J Clin Nurs. 2022;31:3011–3020. doi:10.1111/jocn.16204. 35092116 PMC9787640

[r9] Anwar M, Raziq A, Shoaib M, et al. Exploring nurses’ perception of antibiotic use and resistance: A qualitative inquiry. J Multidiscip Healthc. 2021;14:1599–1608. doi:10.2147/JMDH.S309020. 34234448 PMC8254422

[r10] Edwards R, Drumright L, Kiernan M, Holmes A. Covering more territory to fight resistance: Considering nurses’ role in antimicrobial stewardship. J Infect Prev. 2011;12:6–10. doi:10.1177/1757177410389627. 21532974 PMC3083718

[r11] Hamdy R, Neal W, Nicholson L, Ansusinha E, King S. Pediatric nurses’ perceptions of their role in antimicrobial stewardship: A focus group study. J Pediatr Nurs. 2019;48:10–17. doi:10.1016/j.pedn.2019.05.020. 31200142

[r12] Padigos J, Reid S, Kirby E, Broom J. Knowledge, perceptions and experiences of nurses in antimicrobial optimization or stewardship in the intensive care unit. J Hosp Infect. March 2021;109:10–28. doi:10.1016/j.jhin.2020.12.003. Epub December 5, 2020. PMID: . 33290817

[r13] Catanzaro MT. Antibiotic stewardship for nurses: Using e-learning modules to bridge the education gap. *Antimicrob Steward Healthc Epidemiol*. January 17, 2022;2(1):e7. doi:10.1017/ash.2021.216. PMID: ; PMCID: . 36310815 PMC9614802

[r14] AMR EDUCare. Education on Antimicrobial Resistance in Europe. Retrieved May 25, 2024, from https://www.amreducare.eu/education-on-amr-in-europe/.

[r15] Gotterson F, Buising K, Manias E. Nurse role and contribution to antimicrobial stewardship. An integrative review. Int J Nurs Studies.2020:81:103787. Felix AM , Toffolo SR. Participation of nurses in antimicrobial stewardship programs: An integrative review. *Cogitare Enferm*. 2019;24. 10.1016/j.ijnurstu.2020.103787 33647845

[r16] Barchitta M, Sabbatucci M, Furiozzi F, et al. Knowledge, attitudes and behaviors on antibiotic use and resistance among healthcare workers in Italy, 2019: Investigation by a clustering method. Antimicrob Resist Infect Control. 2021;10:134. doi:10.1186/s13756-021-01002-w. 34507607 PMC8431867

[r17] Ayton D, Watson E, Betts JM, et al. Implementation of an antimicrobial stewardship program in the Australian private hospital system: Qualitative study of attitudes to antimicrobial resistance and antimicrobial stewardship. BMC Health Serv Res. December 20, 2022;22(1):1554. doi:10.1186/s12913-022-08938-8. PMID: ; PMCID: . 36536350 PMC9764684

[r18] World Health Organization. WHO Policy Guidance on Integrated Antimicrobial Stewardship Activities. 2021. https://www.who.int/publications/i/item/9789240025530. Accessed May 25, 2024.

[r19] von Elm E, Altman DG, Egger M, et al. The strengthening the reporting of observational studies in epidemiology (STROBE) statement: Guidelines for reporting observational studies. Lancet. 2007;370:1453–1457. doi:10.1016/S0140-6736(07)61602-X. 18064739

[r20] Finnimore K, Smyth W, Carrucan J, Nagle C. Nurses’ knowledge, practices and perceptions regarding *Clostridioides difficile*: Survey results. Infect Dis Health. 2023;28:39–46. doi:10.1016/j.idh.2022.07.003. 36002370

[r21] Monsees EA, Tamma PD, Cosgrove SE, Miller MA, Fabre V. Integrating bedside nurses into antibiotic stewardship: A practical approach. Infect Contol Hosp Epidemiol. 2019;40(5):579–584. doi:10.1017/ice.2018.362.30786944

[r22] Currie K, Laidlaw R, Ness V, et al. Mechanisms affecting the implementation of a national antimicrobial stewardship programme; multi-professional perspectives explained using normalisation process theory. Antimicrob Resist Infect Control. 2020;9:99. doi:10.1186/s13756-020-00767-w. 32616015 PMC7330968

[r23] Tembo N, Mudenda S, Banda M, Chileshe M, Matafwali S. Knowledge, attitudes and practices on antimicrobial resistance among pharmacy personnel and nurses at a tertiary hospital in Ndola, Zambia: Implications for antimicrobial stewardship programmes. JAC Antimicrob Resist. 2022:4:dlac107. doi:10.1093/jacamr/dlac107. 36226225 PMC9549736

[r24] Di Gennaro F, Marotta C, Amicone M, et al. Italian young doctors’ knowledge, attitudes and practices on antibiotic use and resistance: A national cross-sectional survey. J Glob Antimicrob Resist. 2020;23:167–173. doi:10.1016/j.jgar.2020.08.022. 32971291

[r25] Lévin C, Thilly N, Dousak M, et al. Perceptions, attitudes, and practices of French junior physicians regarding antibiotic use and resistance. Med Mal Infect. 2019;49:241–249. doi:10.1016/j.medmal.2018.09.003. 30266431

[r26] Sánchez-Fabra D, Dyar OJ, Del Pozo JL, et al. Perspective of Spanish medical students regarding undergraduate education in infectious diseases, bacterial resistance and antibiotic use. Enferm Infecc Microbiol Clin (Engl Ed). 2019;37:25–30. doi:10.1016/j.eimc.2017.12.003. 29429753

[r27] Tomczyk S, Taylor A, Brown A, et al. Impact of the COVID-19 pandemic on the surveillance, prevention and control of antimicrobial resistance: A global survey. J Antimicrob Chemother. 2021;76:3045–3058. doi:10.1093/jac/dkab300. 34473285 PMC8499888

[r28] Lucien MAB, Canarie MF, Kilgore PE, et al. Antibiotics and antimicrobial resistance in the COVID-19 era: Perspective from resource-limited settings. Int J Infect Dis. 2021;104:250–254. doi:10.1016/j.ijid.2020.12.087. 33434666 PMC7796801

[r29] Smith DRM, Shirreff G, Temime L, Opatowski L. Collateral impacts of pandemic COVID-19 drive the nosocomial spread of antibiotic resistance: A modelling study. PLOS Med. 2023;20:e1004240. doi:10.1371/journal.pmed.1004240. 37276186 PMC10241372

[r30] Segala FV, Pafundi PC, Masciocchi C, et al. Incidence of bloodstream infections due to multidrug-resistant pathogens in ordinary wards and intensive care units before and during the COVID-19 pandemic: A real-life, retrospective observational study. Infection. 2023;51:1061–1069. doi:10.1007/s15010-023-02000-3. 36867310 PMC9983510

[r31] Pan American Health Organization. Antimicrobial Resistance, Fueled by the COVID-19 Pandemic: Policy Brief November 2021. PAHO, Washington, D.C.; 2021. Retrieved from https://iris.paho.org/handle/10665.2/55864.

[r32] El-Monshed AH, Amr M, Ali AS, Elmasry YM, Zoromba M. Nurses’ knowledge, concerns, perceived impact and preparedness toward COVID-19 pandemic: A cross-sectional survey. Int J Nurs Pract. 2021;27:e13017. doi:10.1111/ijn.13017. 34595803 PMC8646278

[r33] Wendt B, Huisman-de Waal G, Bakker-Jacobs A, Hautvast JLA, Huis A. Exploring infection prevention practices in home-based nursing care: A qualitative observational study. Int J Nurs Stud. 2022;125:104130. doi:10.1016/j.ijnurstu.2021.104130. 34839222

[r34] Rasheed SP, Younas A, Sundus A. Self-awareness in nursing: A scoping review. J Clin Nurs. 2019;28:762–774. doi:10.1111/jocn.14708. 30362645

[r35] Rábano-Blanco A, Domínguez-Martís EM, Mosteiro-Miguéns DG, Freire-Garabal M, Novío S. Nursing students’ knowledge and awareness of antibiotic use, resistance and stewardship: A descriptive cross-sectional study. Antibiotics (Basel). 2019;8:203. doi:10.3390/antibiotics8040203. 31671525 PMC6963445

[r36] Comparcini D, Simonetti V, Segala FV, et al. Nurses’ knowledge, attitudes and practices on the management of clostridioides difficile infection: A cross-sectional study. Antibiotics. 2023;12:529. doi:10.3390/antibiotics12030529. 36978396 PMC10044176

[r37] Chater AM, Family H, Abraao LM, et al. Influences on nurses’ engagement in antimicrobial stewardship behaviours: A multi-country survey using the theoretical domains framework. J Hosp Infect. 2022;129:171–180. doi:10.1016/j.jhin.2022.07.010. 35843415

[r38] Baraka MA, Alsultan H, Alsalman T, Alaithan H, Islam MA, Alasseri AA. Health care providers’ perceptions regarding antimicrobial stewardship programs (AMS) implementation-facilitators and challenges: A cross-sectional study in the Eastern Province of Saudi Arabia. Ann Clin Microbiol Antimicrob. 2019;18:26. doi:10.1186/s12941-019-0325-x. 31551088 PMC6760054

[r39] Roma R. Infermieri Italiani Sempre Più Anziani. Accessed March 11, 2024. https://www.nurse24.it/infermiere/attualita-infermieri/infermieri-italiani-sempre-piu-anziani.html.

[r40] Di Gennaro F, Murri R, Segala FV, et al. Attitudes towards Anti-SARS-CoV2 vaccination among healthcare workers: Results from a national survey in Italy. Viruses. 2021;13:371. doi:10.3390/v13030371. 33652829 PMC7996832

[r41] Segala FV, et al. Perspectives on climate action and the changing burden of infectious diseases among young Italian doctors and students: A national survey. Front. Public Health 2024;12:1382505. doi: 10.3389/fpubh.2024.1382505. PMC11250467 39015393

