# Supplementary material for: Knowledge, Attitudes, and Practices toward Antimicrobial Resistance among Young Italian Nurses and Students: A Multicenter, Cross-Sectional Study
Source: Ann Glob Health. 2024 Jul 22;90(1):46. doi: 10.5334/aogh.4488 (PMC11276540; doi:10.5334/aogh.4488)
Supplement: Supplementary Table. — Distribution of KAP correct answers stratified by job position. [file agh-90-1-4488-s1.pdf]

Supplementary table. Distribution of KAP correct answers stratified by job position.

ITEM	Workers (N=316)	Students (N=525)	Overall (N=841)	p-value
**KNOWLEDGE**				
The optimal intravenous infusion time for Piperacillin/Tazobactam is 3 hours/continuous infusion	152 (48.1%)	212 (40.4%)	364 (43.3%)	**0.034**
Wearing gloves replaces handwashing	302 (95.6%)	510 (97.1%)	812 (96.6%)	0.31
The use of hand sanitizer is equivalent to washing hands with soap and water	204 (64.6%)	369 (70.3%)	573 (68.1%)	0.098
The COVID-19 pandemic has reduced the spread of AMR in our country	248 (78.5%)	369 (70.3%)	617 (73.4%)	**0.011**
It is good practice for all hospitalized patients to be under antibiotic coverage	296 (93.7%)	419 (79.8%)	715 (85.0%)	**<0.001**
In a patient in septic shock, antibiotic administration is one of the actions to be taken during the 'golden hour'	235 (74.4%)	345 (65.7%)	580 (69.0%)	**0.01**
Which of these procedures is NOT part of the 5 fundamental moments of Hand Hygiene: wash your own hands:	208 (65.8%)	279 (53.1%)	487 (57.9%)	**<0.001**
Antibiotic resistance is an expressed property	223 (70.6%)	385 (73.3%)	608 (72.3%)	0.431
KNOWLEDGE SCORE **-** Mean (SD)	6.9 (± 1.5)	6.5 (± 1.4)	6.7 (± 1.4)	**<0.001**
**ATTITUDES**				
How willing are you to support a specific Antibiotic Resistance exam during your academic path?	268 (84.8%)	384 (73.1%)	652 (77.5%)	**<0.001**
How willing are you to attend AMR prevention courses?	292 (92.4%)	451 (85.9%)	743 (88.3%)	**0.006**
How willing are you to create a monitoring network for correct antibiotic administration and report any resistance cases?	291 (92.1%)	437 (83.2%)	728 (86.6%)	**<0.001**
How willing are you to follow indications and procedures that reduce antibiotic resistance?	300 (94.9%)	474 (90.3%)	774 (92.0%)	**0.022**
ATTITUDE SCORE - Mean (SD)	4.6 (± 0.79)	4.3 (± 1.1)	4.4 (± 1.0)	**<0.001**
**PRACTICES**				
I wash my hands before putting on gloves	183 (57.9%)	276 (52.6%)	459 (54.6%)	0.151
I wash my hands after removing gloves	221 (69.9%)	326 (62.1%)	547 (65.0%)	**0.025**
I adhere to the antibiotic administration timeframes	133 (42.1%)	254 (48.4%)	387 (46.0%)	0.088
I use disposable gowns in contact isolations	175 (55.4%)	243 (46.3%)	418 (49.7%)	**0.013**
I notice that contact isolations are not adequately indicated	55 (17.4%)	112 (21.3%)	167 (19.9%)	0.196
I agree that the provided materials (gloves, hand sanitizer, disposable gowns, ROT, etc.) are not readily available	46 (14.6%)	95 (18.1%)	141 (16.8%)	0.217
I ensure that the urine bag in patients with urinary catheters is lifted off the ground	174 (55.1%)	191 (36.4%)	365 (43.4%)	**<0.001**
I dedicate time to educate patients, caregivers, colleagues, support staff about infection risks, mitigation, and AMR management	46 (14.6%)	86 (16.4%)	132 (15.7%)	0.544
**PRACTICES SCORE** - Mean (SD)	4.3 (± 2.1)	4.0 (± 2.4)	4.1 (± 2.3)	0.0591
**KAP SCORE** Mean (SD)	14 (± 3.0)	13 (± 3.2)	13 (± 3.2)	**<0.001**

*The table shows the total number and percentage of correct answers. Bold p value represents a statistical significative variable.
